# Can Targeted Intervention Mitigate Early Emotional and Behavioral Problems?: Generating Robust Evidence within Randomized Controlled Trials

**DOI:** 10.1371/journal.pone.0156397

**Published:** 2016-06-02

**Authors:** Orla Doyle, Edel McGlanaghy, Christine O’Farrelly, Richard E. Tremblay

**Affiliations:** 1 UCD School of Economics and UCD Geary Institute for Public Policy, University College Dublin, Ireland; 2 Clinical Psychology, School of Health in Social Science, University of Edinburgh, Edinburgh, United Kingdom; 3 Centre for Mental Health, Imperial College London, London, United Kingdom; 4 Departments of Psychology and Pediatrics, University of Montreal, Montreal, Canada; 5 UCD School of Public Health, Physiotherapy and Sport Science, University College Dublin, Dublin, Ireland; National Institute of Child Health and Human Development, UNITED STATES

## Abstract

This study examined the impact of a targeted Irish early intervention program on children’s emotional and behavioral development using multiple methods to test the robustness of the results. Data on 164 *Preparing for Life* participants who were randomly assigned into an intervention group, involving home visits from pregnancy onwards, or a control group, was used to test the impact of the intervention on Child Behavior Checklist scores at 24-months. Using inverse probability weighting to account for differential attrition, permutation testing to address small sample size, and quantile regression to characterize the distributional impact of the intervention, we found that the few treatment effects were largely concentrated among boys most at risk of developing emotional and behavioral problems. The average treatment effect identified a 13% reduction in the likelihood of falling into the borderline clinical threshold for Total Problems. The interaction and subgroup analysis found that this main effect was driven by boys. The distributional analysis identified a 10-point reduction in the Externalizing Problems score for boys at the 90^th^ percentile. No effects were observed for girls or for the continuous measures of Total, Internalizing, and Externalizing problems. These findings suggest that the impact of this prenatally commencing home visiting program may be limited to boys experiencing the most difficulties. Further adoption of the statistical methods applied here may help to improve the internal validity of randomized controlled trials and contribute to the field of evaluation science more generally.

***Trial Registration*:** ISRCTN Registry ISRCTN04631728

## Introduction

Evidence suggests that emotional and behavioral problems can be identified as early as age 2 [1] and as many as one in five young children experience difficulties, with higher rates observed among children living in poverty [2]. Although early emotional and behavioral difficulties are transient for some children, for others early distress persists and can represent clinically significant disturbances [3]. While predictions of later severe problems can be made from observational measures at age 3 [4–5], accumulating evidence now suggests that mental health problems may emerge from infancy and the time around children’s second birthday may be a key point of identification [6]. Compromised socio-emotional skills may affect children’s ability to experience, manage, and express emotion, form close and secure relationships, and explore and learn from their environments [7]. Left unattended, frequent and severe difficulties may be associated with poorer outcomes in adolescence and adulthood including later psychopathology, increased delinquency, relationship instability, unemployment, and lower income [4, 8].

Preventing early emotional and behavioral difficulties requires a clear understanding of their causes, which can include genetic, epigenetic, and environmental factors [9–10]. Environmental risks such as chronic poverty [11], abuse or neglect [12], and parental mental health difficulties [8] may undermine parents’ ability to provide children with sensitive and stimulating relationships and environments. Consequently, the quality of parenting is a key mechanism in the development of internalizing and externalizing difficulties [5].

Early intervention programs which address these risks have been proposed as a means of promoting emotional and behavioral wellbeing [13]. Such interventions have been shown to reap benefits across the lifecourse and across multiple outcomes [14]. Given the complexity of factors influencing emotional and behavioral development, adopting a holistic approach to early intervention that targets multiple risk factors may be especially beneficial. In particular, preventative programs which commence antenatally may help to avoid stigmatizion which can arise in later intervention programs.

Home visiting is one form of early intervention which may improve children’s emotional and behavioral health by adopting a preventative and holistic approach. Home visiting is a service delivery strategy shared by programs which otherwise can vary significantly in terms of the type of families served (universal or targeted), the age of the child (pregnancy, birth, or later), service delivery staff (professional or paraprofessional), goals and outcomes (e.g., birth outcomes, immunization, accidents and hospitalization, child maltreatment, parenting practices, and children’s physical, socioemotional, and cognitive development) and program content, intensity and duration. Common to all home visiting programs (HVP), however, is the belief that parents mediate outcomes for their children’s health and development. Often rooted in Attachment [15], Social Learning [16] and Ecological [17] theories, the HVP model prioritizes the provision of warm, sensitive and consistent interactions and positive behavior management, which has been implicated in the development of positive behavior [5]. Thus, shared HVP goals often include enhancing the sensitivity and quality of parenting, promoting parents’ knowledge of child health and development, and enriching parents’ abilities to provide stimulating learning environments. Some HVPs also seek to improve families’ self-sufficiency and reduce the risk for neglect and abuse.

Families are typically supported in reaching these goals through home visits delivered by trained home visitors on a regular basis. Home visitors’ training and roles can also vary, although they are often involved in educating families on child health and development, coaching and modelling positive parenting practices, providing emotional and social support, and improving access to community services [18]. For excellent reviews and detailed information on individual HVPs see [19–23].

Deriving reliable inferences about the impact of HVPs on children’s emotional and behavioral development is complicated by considerable variability in study findings. A systematic review of HVPs identified some significant positive effects for problem behaviors; yet this finding was based on only three studies [24]. Moreover, a Cochrane review reported that there is insufficient high-quality randomized controlled trial (RCT) evidence on the effectiveness of HVPs on children’s socio-emotional functioning [25].

A number of individual HVPs that meet the strict HomeVee criteria (http://homvee.acf.hhs.gov/Default.aspx) identify no impact on emotional and behavioral development at ages 2, 3 or 4 e.g. Nurse Family Partnership [26–27], Early Head Start [28], Family Check-up [29], Comprehensive Child Development Program [30], and Born to Learn [31]. However, there are some exceptions. A Healthy Families Alaska evaluation found a reduction in internalizing and externalizing difficulties as measured by the Child Behavior Checklist [32] at age 2 [33]. An Early Head Start study also reported a treatment effect for internalizing and total behavior problems, but not for externalizing problems, at age 3 [34]. A number of studies of the Family Check Up program also found treatment effects for change and emergence of behavior problems between ages 2 and 4 [35–36]. The limited impact of HVPs on child behavior during toddlerhood, the endpoint for many HVP interventions, does not necessarily imply program ineffectiveness, indeed some HVPs which did not identify effects in toddlerhood, saw the emergence of significant differences in middle childhood [37–38] and adolescence [39–40]. Critically, comparisons across evaluations of different programs (and amongst different evaluations of the same program model) are complicated by issues of variation with respect to the target group, program context, staff, dosage, service user engagement, program flexibility, and fidelity [23].

Methodological discrepancies across studies may also explain the lack of consensus regarding the effectiveness of HVPs on emotional and behavioral problems. There are three issues to consider. First, while some experimental HVP studies benefit from large samples [26, 30], others are constrained by small sample sizes yet utilize large sample test statistics, such as *t* and *F* tests [29, 33, 41]. These methods may result in biased estimates if the outcome data are skewed. Second, attrition is common in longitudinal trials, and while some HVP studies test for differential attrition [30, 35], few adequately account for its effect on treatment outcomes. Third, most HVP studies estimate the average treatment effect, yet such methods may conceal effects that occur at specific intervals of the outcome distribution. Techniques such as quantile regressions, which can test whether HVPs are more or less effective for children with different levels of emotional and behavioral difficulties, are commonly used in economics, yet are infrequently applied in developmental science [42].

The aims of the present study were twofold. First, to investigate the impact of *Preparing for Life* (*PFL*), a community-based HVP in Ireland, on children’s emotional and behavioral development at 24–months using a RCT design. *PFL* is a 5-year program that aims to improve children’s health and development in disadvantaged communities. The ultimate goal of the program is to improve children’s school readiness skills at age 4/5 by intervening during pregnancy and working with families until the children start school. The program adopts a holistic view of school readiness in accordance with best practice which identifies 5 domains of importance—physical health and well-being, socio-emotional development, approaches to learning, language development and emergent literacy, and cognition. The present study examines the evolving impact of the program on one of these domains—socio-emotional development.

The second aim of this study is to address recent calls for developmental scientists to subject their findings to rigorous estimation techniques that are robust to alternative specifications [43]. Specifically, this study utilized methods to counteract some common issues in experimental design which can limit internal validity. The robustness of results derived using traditional methods were tested using an analytic strategy involving inverse probability weighting to address the issue of differential attrition [44] and permutation-based hypothesis testing to estimate treatment effects with small sample data [14], which is particularly pertinent when conducting subgroup analysis [45]. Quantile regressions were also used to supplement the average treatment effect approach and to characterize the distributional impact of the intervention. Despite identified gender differences in the emergence of problem behaviors [46], few studies have investigated the effectiveness of intervention programs by gender. Thus, an interaction and subgroup analysis was also conducted to address calls by Webster-Stratton [47] to “…determine whether there are different behavioral symptoms, developmental pathways, etiological factors, and treatment outcomes for girls and boys” (p. 541).

## Materials and Methods

This study reports on baseline and 24–month data collected in the first RCT of the *PFL* program. The trial was registered with the ISRCTN register, (unique identifier ISRCTN04631728—The evaluation of the *Preparing For Life* early childhood intervention programme, http://www.controlled-trials.com/ISRCTN04631728). The trial was registered post-recruitment rather than prospectively as the trial developers were not aware of this requirement for community-based behavioral interventions at the time the trial began in 2008. All study procedures were approved by the UCD Human Research Ethics Committee, the Rotunda Hospital Ethics Committee, and the National Maternity Hospital Ethics Committee and was conducted and reported in conformity with CONSORT guidelines (see S1 Text: CONSORT Checklist). All participants gave written informed consent before randomization. Information on the design of the trial has been published elsewhere [48] (also see S2 Text: Study Protocol).

### Recruitment

The study enrolled pregnant women from one community in Dublin, Ireland which is classified by the welfare authorities as disadvantaged. The community is characterized by above national average rates of unemployment, school dropout, lone parent households, and public housing. The inclusion criteria included all pregnant women living in the catchment area during the recruitment period, regardless of parity. There were no exclusion criteria. This within-community universal approach was adopted to avoid the stigmatization which may arise in programs with highly selective inclusion criteria. Participation was voluntary and recruitment took place between the 29^th^ of January 2008 and the 4^th^ of August 2010 through two maternity hospitals and in the community. Recruitment and randomization were conducted by the *PFL* recruitment officer.

### Sample size calculation

The sample size was calculated based on a small effect size (ES, standardized difference between group means) for child school readiness skills as identified by a previous meta-analytic study of home visiting programs [49]. Specifically, a mean difference between the intervention and control groups of between 2 and 5 points (depending on the study included in the meta-analysis) on cognitive development scores (average standardized ES = 0.184) was expected. Given this effect size, in order to power the study at the 80% level, based on an alpha level of .05 using a two-tailed t-test, a sample size of approximately 117 families per study arm was required.

### Randomization

An unconditional probability randomization procedure was applied and no stratification or block techniques were used. In total, 233 participants were recruited and 115 were assigned to the intervention group and 118 to the control group. To ensure randomization was not compromised, the computerized procedure generated an automatic email which was sent to the *PFL* program manager and the principal investigator and included the participant’s assignment condition and identification code. Attempts to reassign participants would trigger a second email highlighting any intentional subversion of the randomization process. The population based recruitment rate was 52% based on the number of live births in the community during the recruitment window. A further 22% of eligible participants were not contactable and a further 26% met the *PFL* recruiter or made contact but did not join the program. To identify whether there were systematic differences between eligible participants and eligible non-participants, a socio-demographic profile survey was conducted with a sample of eligible non-participants (n = 102) when their children were 4 years old. The survey asked participants about their current socio-demographic profile and also their profile when they were pregnant at the time of recruitment. An analysis of this data indicated that the eligible non-participants were of a slightly higher socioeconomic status than the participants who joined the program (see S1 Table for a description). This suggests that the program was effective in targeting the families most in need of intervention.

Tests of baseline equivalence between the randomized intervention and control groups using 123 measures found that two groups did not differ on 97% of baseline measures, indicating that the randomization procedure was successful [48]. Significant differences on the remaining 4 baseline measures suggested that the intervention group had greater knowledge of child development and used more community services, while the control group intended to use more childcare and to start childcare at an earlier age. These differences were small in magnitude and consistent with pure chance. At birth, it emerged there were significantly more boys in the intervention group than in the control group (intervention = 51%; control = 35%).

**Fig 1:**
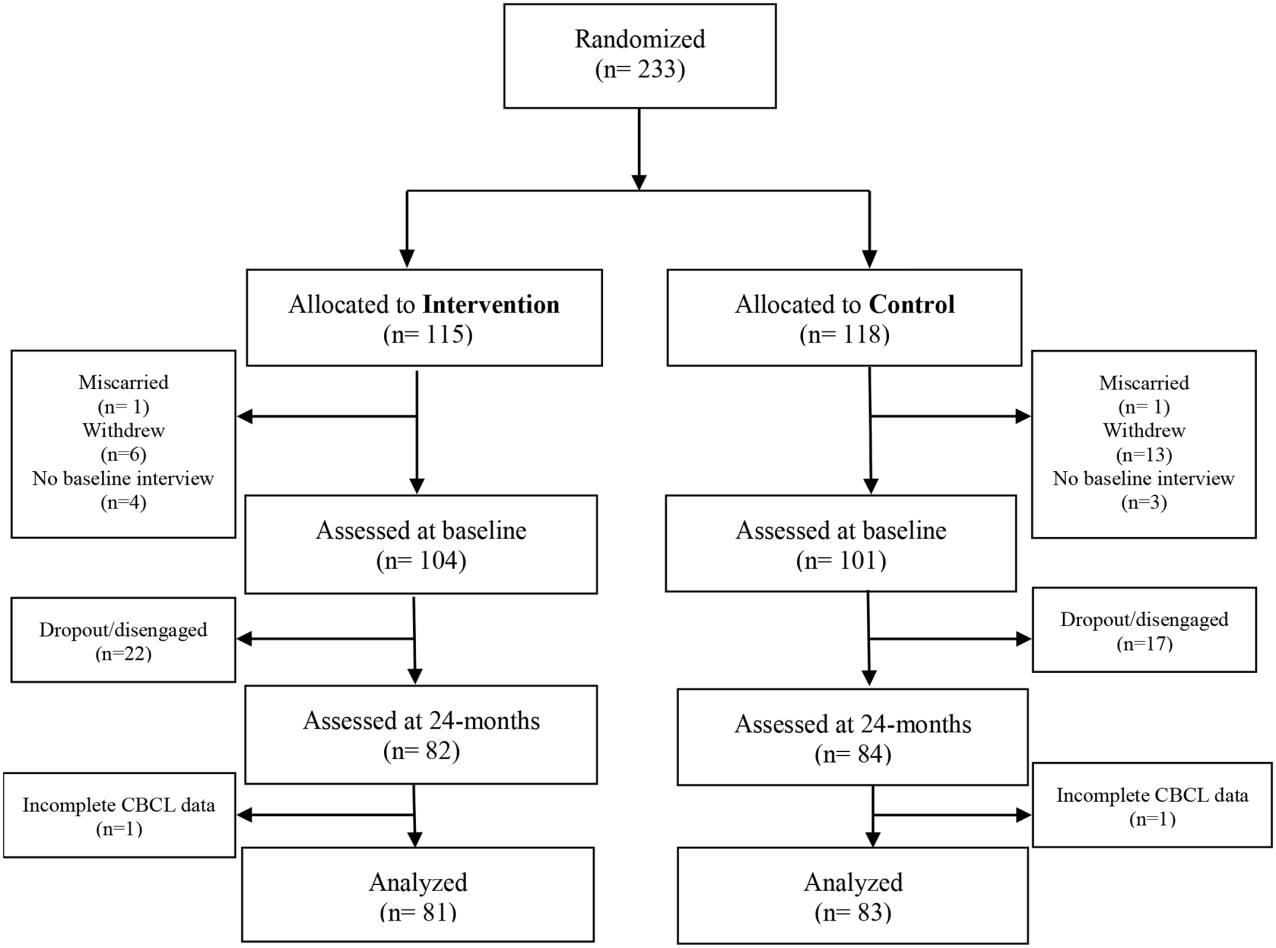
Consort Flow Diagram. **Note:**‘Dropouts’ are those who actively left the study.‘Disengaged’ are those who did not complete the 24-months assessment, but may have re-engaged at some other point.

Of the 233 recruited and randomly assigned participants, 205 (intervention = 104; control = 101) completed the baseline assessment when they were on average 21.5 weeks pregnant (standard deviation (SD) = 7.5 weeks). A 24–month assessment was conducted with 166 participants (intervention = 82; control = 84) when the average child was 24.6 months old (SD = 4.99 weeks). Two participants (intervention = 1; control = 1) were excluded from the final analysis due to missing data on the 24-month outcome. The final estimation sample included 164 participants (intervention = 81; control = 83). Fig 1 shows the participant flow through the trial and the attrition process is described in the results section.

### The intervention

The *PFL* program aimed to improve children’s health and development by intervening during pregnancy and working with families until the children started school. The manualized program was developed by local service providers and community groups in response to evidence that children from the catchment area lagged behind their peers in terms of school readiness skills, including physical health and wellbeing, social and emotional development, approaches to learning, language development and cognition [50]. Grounded in attachment, ecological systems, and social learning theories, the intervention consisted of regular home visits delivered by mentors.

The twice monthly visits, lasting approximately one hour, started during pregnancy and continued until the child started primary school which is typically between the ages of 4 and 5 years old. Participants were prescribed 62 home visits between program entry during pregnancy and when the children were 24-months of age. However, on average, participants received 36 home visits between program entry and 24-months which represents 57% of prescribed visits and is consistent with other HVPs [20]. Table 1 documents prescribed and realized engagement in the program at six monthly intervals. It shows that the number of home visits realized was largely consistent in each period, yet less than prescribed. One factor positively influencing program engagement was the mother’s level of cognitive ability [51].

**Table 1:** Prescribed and Realized Engagement in *PFL* Home Visits.

	Prenatal—birth	Birth– 6 months	6 Months– 12 Months	12 Months– 18 Months	18 Months– 24 Months	Total
Prescribed number of home visits	10	13	13	13	13	62
Prescribed frequency of home visits	Bi-monthly	Bi-monthly	Bi-monthly	Bi-monthly	Bi-monthly	Bi-monthly
Prescribed length of home visits	30mins-2hrs	30mins-2hrs	30mins-2hrs	30mins-2hrs	30mins-2hrs	30mins-2 hrs
Realized number of home visits	6.58 (4.36) 0–21	8.05 (3.84) 0–19	7.62 (3.75) 0–17	7.01 (3.65) 0–21	6.28 (3.04) 0–16	35.54 (15.43) 4–82
% of prescribed home visits realized	71.82 (46.35) 0–350	61.71 (29.46) 0–146	58.47 (28.81) 0–131	53.94 (28.09) 0–162	48.07 (23.24) 0–122	57.25 (23.88) 6–134
Realized length of home visits (mins)	55.50 (18.55) 0–111	58.88 (13.43) 0–91	57.62 (13.40) 0–90	58.17 (15.67) 0–105	58.83 (15.98) 0–89	59.00 (8.77) 40–81
Realized duration of home visits (hours)	6.24 (4.11) 0–18	8.14 (4.22) 0–19	7.52 (3.95) 0–18	7.04 (3.78) 0–19	6.48 (3.36) 0–14	35.42 (16.13) 3–71

Note: The table presents the mean, standard deviation in parentheses, and the minimum and maximum values. These statistics were calculated for intervention participants included in the estimation sample (n = 81).

The aim of the home visits was to support and educate parents on key child rearing issues including the identification of developmental milestones and appropriate parenting practices by fostering a strong parent-mentor relationship [52]. The program is manualized with respect to the overall content that is delivered from pregnancy until the child starts school. Visits were guided by this manual which included a set of 178 *PFL*-developed Tip Sheets based on pre-existing domestic guidelines and succinctly presented best-practice information on pregnancy, parenting, and child development. While some Tip Sheets addressed topics relating to multiple domains of school readiness (e.g., attachment), the focal topic of the majority of Tip Sheets related to physical health and well-being (n = 105), followed by social competence and emotional maturity (n = 60), approaches to learning (n = 30), language (n = 25), and cognitive skills (n = 22). Note that these figures do not sum to 178 as some Tip Sheets are classified in more than one domain.

The home visits typically began with a family update and revision of previous goals, followed by the Tip Sheet(s) for that visit, and goal setting for the next visit. Mentors used role play, modelling, demonstration, coaching, discussion, encouragement, and feedback to deliver the intervention. The model prescribed that all families were given the full curriculum of Tip Sheets as an on-going resource, irrespective of visit uptake. The timing of Tip Sheet delivery and use of the *PFL* strategies varied depended on the child’s age and the mentor’s perception of family need, as well as maternal engagement and learning style. For example, if a particular family already had appropriate dietary patterns in place, the mentors would spend less time on the Tip Sheets related to nutrition; however for other families the mentors may have dedicated multiple visits to the Tip Sheets on nutrition. Thus the program was in keeping with the increasing emphasis on the need for HVPs to offer flexibility with respect to the number, frequency, duration, and focus of visits [23].

The mentors came from a cross-section of different backgrounds with mainly college degrees in education, social care, and youth studies. Each family was assigned the same mentor over the course of the intervention, where possible. The mentors received extensive training prior to program implementation involving a two-day workshop which covered the mentoring role including the evidence-base for mentoring programs; relationships and activities; outcomes and evaluation; policy and practice alignment; and the *PFL* logic model [52]. Training also included 21 other relevant courses including child protection, attachment theory, and team building. Fidelity to the intervention was tracked during monthly 2 hour supervision sessions. Qualitative implementation analysis identified mentors’ self-reported emphasis on and adherence to fidelity as a key component of program effectiveness [53].

#### Promotion of emotional and behavioral development

Children’s emotional and behavioral development was a strong component of the program during the pre-birth to age 2 period and the focal topic of approximately 39 Tip Sheets (21.9%). In pregnancy, mothers were encouraged to undertake activities facilitating rest and relaxation and to think about and to talk to their baby. The focus on the maternal-infant relationship continued post-birth through topics such as communication: “*your newborn baby can look at your face for interaction…he/she can copy your facial expressions*”. A Tip Sheet on attachment, delivered from pre-birth onwards, promoted a close relationship such as: *“Respond to your baby’s crying*, *calmly and consistently*. *This shows your baby that in times of distress*, *you care and can be relied on to be there for him/her”*. Secure base, covered in the first year, highlighted that “*Your child still needs to rely on you to guide and protect him/her…praise and encourage your child when he/she learns and explores*”. Emotional and behavioral wellbeing was also promoted through topics such as self-regulation and confidence, delivered in the first year, followed by self-awareness, fearfulness, temper tantrums, and sibling relationships, delivered in the second year. For example, the temper tantrums Tip Sheet highlighted that “*A child at this stage must try out his/her parent’s limits*, *over and over*, *to understand their rules”* and encouraged parents to use strategies including *“a predictable routine”*. Mentors worked on these topics using role play with the parent, modelling appropriate child interactions, and reinforcement of positive parenting strategies.

#### Common supports

Both the intervention and control groups received annual developmental materials including a baby gym, food utensils, safety items, developmental toys and book packs. Participants in both groups were also encouraged to attend public health workshops on stress management and healthy eating which were already taking place in the community, however relatively few *PFL* participants attended these sessions. For example, only 18 mothers in the intervention group and 9 in the control group participated in the nutrition course. The control group also had access to a support worker who could help them avail of community services, while the mentors provided this function for the intervention group.

### Measures

All data were collected during face-to-face assessments conducted in the participants’ homes by trained researchers who were blind to participants’ treatment assignment and not involved in intervention delivery. Baseline assessments were conducted after randomization yet before the intervention began. Participants received a shopping voucher (€20) as a thank you for completing the assessments.

Emotional and behavioral problems were assessed using maternal reports on the *Child Behavior Checklist for Ages 1½ -5* [32] at the 24-month assessment. The CBCL is a 100 item instrument which generates standardized *T* scores for Internalizing Problems (Cronbach alpha (α) = 0.85), Externalizing problems (α = 0.89), and a Total Problems score (α = 0.95). The high α’s indicated the reliability of the CBCL instrument within this sample. In addition, for each scale the borderline cutoff *T* score > 60 was used to index children with more significant emotional and behavioral problems. Missing data for individual items were imputed using the mean plus a random residual value and was approved by the instrument’s developer [54]. If more than 8 items were missing, participants were excluded from the analysis (n = 2).

### Analysis

All analyses were conducted using STATA MP 12. The study adopted an intention-to-treat strategy and two-tailed *p*-values were reported throughout. OLS regression models were estimated for the *T* scores and logistic regressions were estimated for the cutoff scores. Both measures were utilized as previous HVP studies report either the former e.g., [33] or the latter e.g., [40]. To account for potential bias due to differential attrition [44, 55], an inverse probability weighting (IPW) technique was applied. This involved three steps. First, 116 bivariate t-tests were conducted to examine the individual baseline factors associated with participation in the 24–month assessment. The attrition analysis was restricted to participants who completed a baseline assessment. IPW is based on the assumption that the outcome is independent of the missing data pattern, conditional on treatment assignment and observable baseline characteristics. Variables which were statistically significant in the bivariate tests (at the 10% level in a two-tailed test), had no missing data, and were not collinear with any other characteristic were retained. Second, the significant variables were included in a logistic model which was used to calculate the predicted probability of completing the 24–month assessment for each participant. Separate logistic models were conducted for the intervention and control groups as differential attrition processes may exist. Third, the inverse of these predicted probabilities were then applied as weights in the outcomes analysis. Applying these weights ensured that a larger weight was given to participants that were underrepresented in the sample due to attrition i.e. those who completed the 24–month assessment yet had characteristics which were similar to those who dropped out. One participant who participated in the 24-month assessment, yet did not participate in the baseline assessment, was assigned the average IPW-weight.

To determine whether the program had differential effects by gender, the above models were re-estimated controlling for treatment status, gender, and an interaction between the two. Any interactions between gender and treatment assignment which trended towards significance were further investigated via subgroup analysis. As the sample size of the subgroup analysis was relatively small, thus increasing the chances that the data were not normally distributed i.e., skewed, the robustness of these results was tested using permutation testing [56]. This method is advantageous as it does not depend on distributional assumptions and thus is more suitable when the sample size is small. Simulation studies have found that permutation testing performs better than *t* tests, particularly if the degree of skewness is correlated with the size of the treatment effect [57].

A permutation test is based on the assumption of exchangeability under the null hypothesis. This means that if the null hypothesis is true i.e., the program has no impact on the children’s outcomes, taking random permutations of the treatment indicator by moving participants between the intervention and control groups, does not change the results. Permutation tests work as follows: first, the mean outcomes of the intervention and control groups are compared and a test statistic is calculated. Second, the data are repeatedly shuffled so that the treatment assignment of some participants is switched between groups and a test statistic is calculated each time to examine whether the observed result is likely to occur by chance. Third, the *p*-value for the permutation test is computed by examining the proportion of permutations that have a test statistic more extreme than the observed test statistic. If the proportion is small, we know that the original statistic is an unlikely outcome. Permutation tests based on 100,000 replications were used. A full description of the IPW and permutation methods can be found in these studies [58–59].

Most intervention studies estimate the average treatment effect, however, the magnitude of an effect may vary across the distribution of the outcome. This study used the quantile regression method [60] to examine whether the intervention had heterogeneous effects at different points in the distribution of the continuous CBCL scores. Quantile regression estimates the conditional distribution by dividing the cumulative distribution of the outcome into defined intervals. Importantly, quantile regression makes no assumptions regarding the distribution of the residual, which helps avoid biased estimates if the outcome exhibits skewness, as is the case with the CBCL [61]. In line with Petscher and Logan [42] and also Huston et al. [62] program impacts were estimated at the 10^th^, 25^th^, 50^th^ (median), 75^th^, and 90^th^ percentiles of CBCL scores, as this provided upper and lower bound points, as well as values corresponding to the interquartile range, yet was not overly specific given the sample size. This allowed us to determine whether the averaged OLS regression results concealed larger impacts at different ends of the distribution. The quantile models were also estimated including gender by treatment status interactions.

While the OLS, logistic, permutation, and IPW analyses were pre-specified, the quantile analysis was exploratory. As a sensitivity test, all analyses were re-estimated while controlling for the 4 significant differences between the intervention and control groups at baseline. These results are reported in S2 Table.

## Results

### Sample characteristics and attrition

Table 2 reports selected baseline characteristics of the estimation sample. On average, mothers were between 25 and 26 years old when they joined the program, typically during the 20^th^ week of pregnancy. Approximately half were first time mothers and lived in public housing, while one-third had not completed a second level education and over 40% were unemployed. A quarter of the sample had a diagnosed mental health condition, and almost half smoked during pregnancy. A detailed description of the original *PFL* sample was reported here [48]. Twenty-nine percent of recruited participants did not participate in the 24–month assessment due to attrition. Attrition includes participants who 1) voluntarily dropped out of the study prior to 24–months, 2) involuntarily dropped out of the study prior to 24–months due to miscarriage, still birth or moved region/country, and 3) wave non-response i.e. did not engage in the 24–month assessment yet re-engaged with the evaluation at later waves. Exit questionnaires revealed that the primary reason for voluntary dropout was ‘*the program may take/did take up too much of my time*’.

**Table 2:** Baseline maternal characteristics of estimation sample.

	*M*_intervention_ (*SD*)	*M*_control_ (*SD*)	*p-*value^i^
Weeks pregnant at program entry	21.86 (7.99)	21.34 (6.93)	0.656
Age	25.90 (5.88)	25.57 (6.28)	0.731
Married (%)	0.16 (0.37)	0.17 (0.38)	0.861
Partnered (including married) (%)	0.79 (0.41)	0.81 (0.39)	0.665
Living with parent(s) (%)	0.54 (0.50)	0.46 (0.50)	0.308
First time mother (%)	0.52 (0.50)	0.46 (0.50)	0.482
Low education (%)	0.30 (0.46)	0.35 (0.48)	0.434
Employed (%)	0.42 (0.50)	0.41 (0.50)	0.947
Saves money regularly (%)	0.48 (0.50)	0.55 (0.50)	0.390
Resides in social housing (%)	0.53 (0.50)	0.55 (0.50)	0.819
IQ (WASI)	83.25 (12.35)	81.65 (12.16)	0.406
Prior physical health condition (%)	0.77 (0.43)	0.63 (0.48)	0.068
Prior mental health condition (%)	0.27 (0.45)	0.26 (0.44)	0.822
Smoking during pregnancy (%)	0.51 (0.50)	0.46 (0.50)	0.585
Drinking during pregnancy (%)	0.27 (0.45)	0.27 (0.45)	0.962
Drugs ever used (%)	0.14 (0.34)	0.12 (0.33)	0.792
Vulnerable attachment (VASQ)	18.00 (3.90)	17.59 (3.86)	0.496
Positive parenting attitudes (AAPI)	5.24 (1.24)	5.26 (1.31)	0.925
Self-efficacy (Pearlin)	2.80 (0.60)	2.89 (0.61)	0.367
Self-esteem (Rosenberg)	12.98 (2.62)	12.71 (2.90)	0.537
Knowledge of infant development (KIDI)	72.52 (7.11)	70.57 (8.31)	0.110

Note: N = 163 (intervention 81; control 82). Note that one participant who did participate in the 24-month assessment, did not complete a baseline survey, thus the sample size for the baseline descriptives is 163 rather than 164. ‘M’ indicates the mean. ‘SD’ indicates the standard deviation.

^i^ two-tailed p-value from either a t-test for continuous outcomes or a chi-squared test for binary outcomes.

‘First time mother’ refers to the proportion of participants who had no previous children when entering the *PFL* program. ‘Low education’ represents participants who left school after they completed a statewide examination at age 15 to 16 years. ‘Saves money’ regularly refers to the proportion of participants who claimed to save money on a regular basis. IQ was measured 3 months post-birth using the Wechsler Abbreviated Scale of Intelligence (WASI). Physical health condition indicates whether the mother has ever been diagnosed with any of 22 listed conditions. Mental health condition indicates whether the mother has ever been diagnosed with any 8 listed mental health conditions. Smoking during pregnancy represents participants who said they were currently a smoker when asked during pregnancy. Drinking during pregnancy represents participants who said they drank any alcohol during pregnancy. Drugs ever used represents participants who claimed to have taken any drug from a list of 15 at any point in their lives. The Vulnerable Attachment Style Questionnaire (VASQ) measures the respondents' interactions and dependence on other people. Scores above 15 are indicative of depressive disorders. The Adult Adolescent Parenting Inventory (AAPI) measures approaches to parenting and provides an indicator of the endorsement of abuse/neglect. Higher scores indicate a high risk of abuse/neglect. The Pearlin Self-Efficacy scale ranges from zero to four with higher scores indicating higher self-efficacy. The Rosenberg self-esteem scale ranges from zero to 18 with higher scores indicating more maternal self-esteem. The Knowledge of Infant Development (KIDI) score represents the percentage of correct responses to questions relating to child development milestones. Higher scores indicate more knowledge of infant development.

While the rate of attrition was equal for both groups (intervention = 29%, control = 29%) a bivariate analysis found 9.5% of baseline measures significantly predicted attrition from the intervention group and 15% of baseline measures predicted attrition from the control group. As this analysis suggested some evidence of differential attrition, it was necessary to account for these differences using IPW.

### Impact on emotional and behavioral functioning

Table 3 presents descriptive statistics for the CBCL outcomes. Panel A in Table 4 presents the unweighted and IPW-weighted main effects. The weighted coefficients were similar in magnitude and precision to the unweighted estimates, thus interpretation focuses on the IPW-adjusted models as they provide the most accurate representation of the program’s impact for the original sample. All three coefficients on treatment status were negative, implying that the intervention group had fewer emotional and behavioral problems on average, as measured by the CBCL *T* scores, relative to the control group. However, none of these effects were statistically significant. Panel A also presents the marginal effects for the CBCL cutoff scores. While all three were negative, only the Total Problems cutoff score was significantly associated with treatment status. The estimated marginal effect implies that intervention participants were 13% less likely to be classified as being in the cutoff category for Total Problems.

**Table 3:** Distribution of unweighted CBCL outcomes.

	Total^a^		Boys^b^		Girls^c^		Intervention v Control			
	*M* (*SD*)	*Min-Max*	*M* (*SD*)	*Min-Max*	*M* (*SD*)	*Min-Max*	*M*_INT_ (*SD*)	*Min-Max*	*M*_CON_ (*SD*)	*Min-Max*
*CBCL domains*										
Total Problems	46.53 (10.05)	28–72	46.51 (10.15)	28–72	46.54 (10.02)	28–72	45.79 (9.16)	28–63	47.25 (10.84)	28–72
Internalizing Problems	46.20 (10.22)	29–73	46.43 (10.39)	29–73	46.03 (10.14)	29–73	45.84 (10.13)	29–65	46.55 (10.35)	29–73
Externalizing Problems	45.98 (9.40)	29–68	45.93 (9.87)	29–68	46.01 (9.09)	29–63	45.78 (8.56)	29–63	47.25 (10.84)	29–68
	Total^a^		Boys^b^		Girls^c^		Intervention v Control			
*CBCL domains cutoff scores*	*N* (*%*)		*N* (*%*)		*N* (%)		*N*_INT_ (*%*)		*N*_CON_ (%)	
Total Problems Cutoff	17 (10.37%)		5 (7.14%)		12 (12.77%)		4 (4.94%)		13 (15.66%)	
Internalizing Problems Cutoff	19 (11.59%)		10 (14.29%)		9 (9.57%)		9 (11.11%)		10 (12.05%)	
Externalizing Problems Cutoff	16 (9.76%)		8 (11.43%)		8 (8.51%)		7 (8.64%)		9 (10.84%)	

Note:

^a^ n = 164 (intervention 81; control 83),

^b^ n = 70 (intervention 41; control 29),

^c^ n = 94 (intervention 40; control 54).

‘M’ = mean. ‘SD’ = standard deviation. ‘N’ = sample size.

**Table 4:** Impact of *PFL* on emotional and behavioral functioning—Main and interaction effects.

	*UW*	*IPW*	*UW*	*IPW*	*UW*	*IPW*
**Panel A: Main Effect Models**	CBCL Internalizing Problems		CBCL Externalizing Problems		CBCL Total Problems	
Treatment	-0.71 (1.60)	-1.30 (1.81)	-0.39 (1.47)	-1.33 (1.61)	-1.46 (1.57)	-2.36 (1.77)
	CBCL Internalizing Problems Cutoff		CBCL Externalizing Problems Cutoff		CBCL Total Problems Cutoff	
Treatment	-0.09 (0.05)	-0.01 (0.06)	-0.02 (0.05)	-0.04 (0.05)	-0.10* (0.04)	-0.13** (0.05)
**Panel B: Interaction Effect Models**	CBCL Internalizing Problems		CBCL Externalizing Problems		CBCL Total Problems	
Treatment	-0.58 (2.15)	-0.92 (2.44)	-0.19 (1.98)	-1.24 (2.13)	-0.86 (2.11)	-1.62 (2.39)
Gender (Boy)	0.79 (2.37)	2.28 (2.97)	0.22 (2.18)	0.89 (2.74)	0.99 (2.33)	2.15 (2.97)
Treatment*Gender	-0.52 (3.30)	-1.46 (3.93)	-0.46 (3.04)	-0.46 (3.52)	-1.50 (3.23)	-2.11 (3.81)
	CBCL Internalizing Problems Cutoff		CBCL Externalizing Problems Cutoff		CBCL Total Problems Cutoff	
Treatment	0.06 (0.07)	0.07 (0.08)	0.07 (0.06)	0.06 (0.06)	-0.05 (0.06)	-0.07 (0.07)
Gender (Boy)	0.11 (0.06)	0.15* (0.08)	0.11* (0.05)	0.13* (0.06)	0.02 (0.07)	0.03 (0.10)
Treatment*Gender	-0.14 (0.09)	-0.18 (0.11)	-0.19* (0.08)	-0.19* (0.09)	-0.12 (0.10)	-0.13 (0.11)

Note: n = 164 (intervention 81; control 83). The main effect models reported in Panel A and the interaction effect models reported in Panel B represent different models. *UW*: Unweighted results. *IPW*: Inverse Probability Weighted results. Regression coefficients and standard errors (in parentheses) are reported for the continuous scores. Marginal effects and standard errors (in parentheses) are presented for the binary variables. A logistic model for the Total Problems cutoff score in the interaction analysis could not be estimated as none of the male children in the intervention group reached the cutoff, instead a linear probability model was estimated via OLS.

*p<0.05;

**p<0.01.

### Impact on emotional and behavioral functioning by gender

Panel B of Table 4 presents the unweighted and IPW-weighted results from multivariate models testing for gender by treatment status interactions. All three coefficients on gender in the CBCL *T* score models were positive, implying that boys had more emotional and behavioral problems on average relative to girls. However, none of these effects were statistically significant. The interactions between treatment status and gender were all negative, implying that the program had a larger impact on boys, yet none of the interactions were significant. Panel B also presents the marginal effects for the CBCL cutoff scores. A linear probability model (LPM) was used for the Total Problems cutoff score as a logistic model could not be estimated as none of the intervention boys reached the cutoff. The results show that boys were significantly more likely to be in the cutoff category for both Internalizing and Externalizing Problems than girls. There was a significant gender by treatment status interaction for the Externalizing cutoff, and the Internalizing cutoff trended towards significance, indicating that the program had a larger impact on boys relative to girls.

Table 5 explored the nature of these interactions by conducting subgroup analysis using logistic regression and permutation testing. Only the cutoff scores were considered as there was no evidence of significant interactions between treatment status and gender for the continuous scores. Again LPM was used for the Total Problems cutoff score for boys. The *p*-values resulting from the logistic regressions and permutation testing were very similar, suggesting that the distributional assumptions imposed by the traditional tests were not overly restrictive for the current sample. Similarly, the unadjusted and IPW-adjusted results were broadly equivalent. Overall, there were no statistically significant differences between the intervention and control groups on the three CBCL cutoff scores for girls. There was a statistically significant effect on the Total Problems cutoff score for boys, such that a lower proportion of boys in the intervention group were classified as being in the cutoff category compared to boys in the control group. There was also a trend towards a lower proportion of boys in the intervention group falling into the Externalizing Problems cutoff category. The effect sizes based on Cramer’s Phi were classified as small to medium.

**Table 5:** Impact of *PFL* on emotional and behavioral functioning—Treatment effects by gender.

	Boys^a^					Girls^b^				
CBCL Cutoff Scores	*Log*.*p-value*^1^	*IPW adj*. *Log*.*p-*value^2^	*Perm p-*value^3^	*IPW adj*. *perm p-*value^4^	ES	*Log*. *p-value*^1^	*IPW adj*. *Log*. *p-*value^2^	*Perm*. *p-*value^3^	*IPW adj*. *perm p-*value^4^	ES
Internalizing Problems Cutoff	0.207	0.173	0.268	0.208	0.15	0.412	0.335	0.388	0.443	0.09
Externalizing Problems Cutoff	0.058	0.071	0.078	0.098	0.24	0.245	0.367	0.254	0.254	0.12
Total Problems Cutoff	0.005**	0.023**	0.005**	0.001**	0.33	0.492	0.325	0.447	0.083	0.07

Note:

^a^ n = 70 (intervention 41; control 29),

^b^ N = 94 (intervention 40; control 54).

Perm = permutation test. ‘ES’ = Cramer’s Phi effect size.

^1^ two-tailed p-value from a logistic regression. For boys Total Problems cutoff a LPM model was fitted rather than a logistic regression as treatment status was a perfect predictor of being in the cutoff category.

^2^ two-tailed p-value from a logistic regression (again a LPM model was used for boys Total Problems cutoff) applying inverse probability weights.

^3^ two-tailed p-value from a permutation test with 100,000 replications.

^4^ two-tailed p-value from a permutation test with 100,000 replications applying inverse probability weights.

* p <.05,

** p <.01 level.

### Distributional impact on emotional and behavioral functioning

Table 6 presents results from the quantile regressions. There were no positive treatment effects in the main effect models at any interval. A significant negative treatment effect was identified for Externalizing Problems for children at the 10^th^ percentile. This suggests that the intervention group had more emotional and behavioral difficulties than the control group amongst children exhibiting the lowest levels of problems. In the interaction models, a consistent pattern emerged at the 90^th^ percentile. Boys had significantly more Total Problems and Externalizing Problems than girls (Internalizing Problems trended towards significance). In addition, the significant interaction term on Externalizing Problems indicated that the program was more effective for boys in the intervention group (for Internalizing Problems and Total Problems the interactions trended towards significance). Specifically, the treatment resulted in a 10-point reduction in the Externalizing Problems score for boys at the 90^th^ percentile. There were no treatment effects for girls at any interval. These results suggest that the intervention was most effective for boys with the highest levels of difficulties.

**Table 6:** Quantile regression results of the distributional impact of *PFL* on emotional and behavioral functioning.

CBCL Quartile	0.10	0.25		0.50	0.75	0.90	0.10	0.25	0.50	0.75	0.90
		Main Effect Models						Interaction Effect Models			
Internalizing Problems											
Treatment	-4.0 (2.23)	-4.0 (2.75)	0.0 (1.98)		-2.0 (2.89)	0.0 (3.94)	-4.0 (2.98)	0.0 (3.49)	0.0 (2.14)	0.0 (3.94)	3.0 (3.09)
Gender (Boys)	~	~	~		~	~	0.0 (3.29)	0.0 (3.86)	-2.0 (2.36)	1.0 (4.35)	6.0 (3.41)
Treatment*Gender	~	~	~		~	~	4.0 (4.57)	-4.0 (5.36)	4.0 (3.29)	-3.0 (6.04)	-9.0 (4.75)
Externalizing Problems											
Treatment	3.0* (1.48)	2.0 (2.06)	1.0 (1.99)		-3.0 (2.20)	-4.0 (3.08)	3.0 (2.36)	1.0 (2.89)	0.0 (2.89)	-4.0 (3.08)	1.0 (2.68)
Gender (Boys)	~	~	~		~	~	-3.0 (2.60)	-2.0 (3.18)	-2.0 (3.19)	-3.0 (3.40)	7.0* (2.96)
Treatment*Gender	~	~	~		~	~	3.0 (3.62)	1.0 (4.43)	3.0 (4.44)	4.0 (4.72)	-10.0* (4.11)
Total Problems											
Treatment	-1.0 (2.21)	1.0 (2.06)	0.0 (2.53)		-2.0 (2.48)	-1.0 (3.73)	0.0 (2.45)	1.0 (2.56)	0.0 (3.38)	-1.0 (3.73)	-2.0 (2.57)
Gender (Boys)	~	~	~		~	~	1.0 (2.71)	-1.0 (2.83)	-1.0 (3.73)	2.0 (4.11)	6.0* (2.84)
Treatment*Gender	~	~	~		~	~	0.0 (3.76)	-1.0 (3.93)	-2.0 (5.18)	-4.0 (5.71)	-7.0 (3.94)

Note: n = 164 (intervention 81; control 83). Coefficients and standard errors (in parenthesis) from quantile regression models.

* p <.05,

** p <.01 level.

## Discussion

This study investigated the early impact of an Irish home visiting program and demonstrated the importance of using multiple estimation techniques to test the robustness of results arising from experimental trials. These methods provide greater confidence that the *PFL* treatment effects for emotional and behavioral difficulties at 24 months were limited to boys with the greatest level of problems, while no effects were found for girls. The results were robust to differential attrition, small sample size, and distributional effects. The lack of effects for the continuous measures of total, externalizing, and internalizing problems at 24 months suggests that the intervention was not effective at reducing behavioral problems overall, however it is possible, as seen in other HVPs [38], that effects may emerge later in childhood as children start to exhibit increasing behavioral difficulties.

There was one main treatment effect which implies that children in the intervention group demonstrated a 13% reduction in the likelihood of falling into the borderline clinical threshold for Total Problems. Further investigation revealed that this effect was comprised of a positive treatment effect for boys with the greatest level of emotional and behavioral difficulties and a counterintuitive negative treatment effect for children exhibiting the lowest levels of externalizing problems. One conceivable explanation for this negative effect is that intervention parents whose children had relatively few behavioral issues may have been made more aware of their child’s behavioral profile due to the mentoring. From a practical perspective, this 3 point increase in Externalizing Problems for children with the least behavioral difficulties is unlikely to be substantive for child and family wellbeing. The positive treatment effects identified at the 90^th^, but not at the 75^th^, percentile, corresponds to the treatment effects observed at the CBCL borderline threshold that falls between these two intervals. This demonstrates the added benefit of conducting quantile regressions to uncover treatment effects which may otherwise be concealed using average treatment effect methods.

Overall the findings are in keeping with the mixed evidence for the effectiveness of HVPs on child emotional and behavioral functioning in toddlerhood. Nonetheless, while the treatment effects were limited for the entire sample, the effects observed for children who did benefit from the intervention represent clinically meaningful differences. A 10-point reduction in the Externalizing Problems score for boys at the 90^th^ percentile, which is a full standard deviation, is likely to represent a material improvement in the day-to-day experiences of toddlers and their parents. These effects may be attributed to the emphasis in HVPs on enhancing attachment and parent-child interactions from pregnancy onwards. These mechanisms may have either helped prevent the development of behavior problems or provided parents with appropriate coping strategies in the incident of behavioral problems. That the children most at risk of developing behavioral problems benefitted most from intervention is in keeping with the differential susceptibility model [63] which posits that children who are vulnerable to behavioral difficulties due to genetic or temperamental factors are also more vulnerable to risk factors, and by extension, more likely to thrive in a nurturing environment. The results also accord with Shaw et al’s research [64] on low income children, which found that 2 year old boys with the highest disruptive behaviors showed the greatest persistence in behavioral problems at school age, and crucially, persistence was associated with a more ‘rejecting’ style of parenting behavior.

While most HVP studies do not analyze gender differences despite calls to the contrary [47], those that do typically find no differences [30]. As children mature, it appears that girls may derive more benefits from early intervention than boys in relation to cognitive abilities, education, employment, and criminal behavior [14], however a recent study identified long-term health effects for boys [65]. The literature provides little guidance on how gender interacts with children’s differential susceptibility. However, the finding that boys were most at risk in terms of total behavior problems supports previous research demonstrating that preschool boys are more likely to have clinically significant total behavior problems compared to girls [66].

Clinically, the identification of preventive interventions that mitigate behavioral disturbances is a key objective for psychology and psychiatry [67]. These results provide some support for the hypothesis that intensive HVPs initiated during pregnancy may be an effective vehicle for reducing the incidence of emotional and behavioral problems for boys at age two. Yet the modest nature of the current findings should not be overstated and further research is needed to determine whether the treatment effects identified here are sustained over time and across gender. While the results suggest that the *PFL* intervention had a limited impact on children’s development at age 2, it is possible that there may be continued effects as the children develop and progress through the intervention and beyond, as has been demonstrated in other HVPs [40] or equally, that the effects observed here do not persist. Latent class analysis may also be useful in tracking the patterns of early responders and non-responders over future outcome intervals. Until such studies have been conducted, more refined targeting of the *PFL* program remains premature. Indeed, given the holistic nature of HVPs, their antenatal commencement, and the difficulty in anticipating future levels of behavioral need, further targeting is likely to be complex. It is possible that decision flowcharts could be used to guide Tip Sheet administration and sequencing, similar to modular therapies used in clinical settings [68]. However, this concept would require development and rigorous assessment.

As with all single-site community interventions with voluntary recruitment, the results may not be generalizable to other sites or different populations, however the manualized nature of the intervention allows for retesting of the *PFL* program in other settings. The lack of observational data on the quality of implementation is also a significant limitation, although fidelity to the program manual has been noted elsewhere [53]. The use of maternal reports of child behaviour, which may be subject to bias, is also noteworthy, especially in experimental trials. Yet parental report is frequently used to assess emotional and behavioral problems in preschool samples and the CBCL offers strong psychometric properties [2]. Nonetheless, future research should consider the use of a second informer, clinical interview/observation measures, or information on psychological service referral. Another potential issue which may biased the results is contamination or spillover effects across the intervention and control groups. As the intervention was operating in a very small community and participants were randomly assigned at the individual level, it is possible that the intervention group engaged in cross-talk or intentionally or unintentionally shared their parenting materials, information, strategies, or advice which they received from their mentors, with the control group. However, as reported elsewhere [59], a series of contamination questions devised to measure cross-talk and information flows between the two groups found no evidence that contamination was likely to confound the results.

One contribution of this study is the use of multiple statistical methods which may have applicability to other experimental trials. The methods applied here may help counteract problems which often arise in RCTs such as differential attrition, small sample size data, and heterogeneous effects. The equivalence of the unweighted and the IPW-weighted results suggest that differential attrition did not materially bias the unweighted findings. This suggests that while some participants did drop out of the study, the types of participants who dropped-out of intervention and control groups were largely similar. However, given the extent to which non-random attrition may undermine internal validity, it is important for RCT studies to test and adjust for its presence [69]. Similarly, the logistic regression methods and the permutation methods generated similar inferences for the subgroup analysis, implying that the distributional assumptions necessitated by the traditional tests were not overly restrictive. Yet this method may be particularly advantageous in other samples. It is important to note that the use of permutation testing cannot be used to justify a small sample or an unpowered design. Permutation testing addresses the non-normality issue, which is typically more common in small samples. Thus, an appropriately powered study with a small sample and normally distributed data may not benefit from the use of permutation testing. Limitations of the IPW and permutation methods include the need for high computational power and user written code, however, with greater usage, these methods should become easier to implement in existing statistical packages. Finally, it is important to note that the few statistically significant findings may be a result of Type I errors given the number of different tests conducted. However, the various methods also revealed a certain level of consistency across findings, such that in the logistic, permutation, and quantile regressions, the results were restricted to boys within the clinical range.

Another strength of this study was the move beyond examining the average treatment effect. The ability to identify which part of the distribution is most affected by the intervention may be informative regarding future targeting and program roll-out. However, the use of quantile regression in experimental developmental studies remains new and further work is needed to reach agreed conventions on best practice [42]. In addition, the sample size included at different parts of the quantile distribution were relatively small, thus caution should be exercised when interpreting these results.

In conclusion, this study found some evidence that a prenatally commencing early intervention program had positive effects on the emotional and behavioral functioning for boys experiencing the most difficulties. The wider use of the methods adopted here may help to improve the internal validity of RCTs and consolidate knowledge which has significant translational value for improving child development and mental health. While we acknowledge that addressing these specific methodological issues is not a panacea for the challenges in identifying which HVPs work, for whom and under what conditions, the methods presented here represent a step forward in contributing to the HVP literature, as well as contributing to the field of evaluation science more generally.

## Supporting Information

S1 Data (XLS)

S1 Table Comparison of Participants and Eligible Non-participants at Baseline. (DOCX)

S2 Table **Table A)** Impact of *PFL* on emotional and behavioral functioning—Main and interaction effects controlling for baseline differences. **Table B)** Impact of *PFL* on emotional and behavioral functioning—Treatment effects by gender controlling for baseline differences. **Table C)** Quantile regression results of the distributional impact of *PFL* on emotional and behavioral functioning controlling for baseline differences.
(DOCX)

S1 Text CONSORT Checklist. (DOC)

S2 Text Study Protocol. (DOC)
