# Supplementary material for: Can Targeted Intervention Mitigate Early Emotional and Behavioral Problems?: Generating Robust Evidence within Randomized Controlled Trials
Source: PLoS One. 2016 Jun 2;11(6):e0156397. doi: 10.1371/journal.pone.0156397 (PMC4890862; doi:10.1371/journal.pone.0156397)
Supplement: S1 Table — (DOCX) [file pone.0156397.s002.docx]

**S1 Table**

**Comparison of Participants and Eligible Non-participants at Baseline**

|  | Total *N*  *(PFL/Comp.)* | *PFL*  Mean (SD) | *Comp.*  Mean (SD) | Two-sided *p^i^* |
| --- | --- | --- | --- | --- |
| *Demographics* |  |  |  |  |
| Gender of study child – Girl (%) | 301  (199/102) | 0.54  (0.50) | 0.47  (0.50) | 0.232 |
| Number of older siblings the study child has | 286  (210/76) | 0.93  (1.24) | 0.99  (1.05) | 0.729 |
| Mothers Age in 2000 | 325  (226/99) | 16.26  (5.92) | 17.20  (6.31) | 0.203 |
| Mothers Age when had first child | 311  (210/101) | 21.65  (4.16) | 23.53  (5.39) | **0.003** |
| Teen mother when had first child (%) | 311  (210/101) | 0.35  (0.48) | 0.29  (0.46) | 0.247 |
| First-time mother when had study child (%) | 312  (210/102) | 0.51  (0.50) | 0.55  (0.50) | 0.578 |
| Mother eligible for free medical care (%) | 306  (210/96) | 0.63  (0.48) | 0.47  (0.50) | **0.009** |
| *Maternal Education* |  |  |  |  |
| Age left full-time education | 282  (191/91) | 17.41  (2.78) | 17.81  (2.08) | 0.187 |
| Finished full-time education (%) | 253  (152/101) | 0.86  (0.35) | 0.90  (0.30) | 0.349 |
| Leaving Cert education or higher (%) | 312  (210/102) | 0.46  (0.50) | 0.59  (0.50) | **0.037** |
| Left school before the age of sixteen (%) | 282  (191/91) | 0.19  (0.39) | 0.04  (0.21) | **0.000** |
| *Maternal Employment when Pregnant* |  |  |  |  |
| Employed (%) | 306  (210/96) | 0.39  (0.49) | 0.67  (0.47) | **0.000** |
| Engaged in skilled work (%) | 133  (76/57) | 0.71  (0.46) | 0.61  (0.49) | 0.256 |
| *Maternal Relationship Status when Pregnant* |  |  |  |  |
| Married (%) | 306  (210/96) | 0.16  (0.37) | 0.24  (0.43) | 0.103 |
| Partner (%) | 306  (210/96) | 0.81  (0.39) | 0.85  (0.36) | 0.385 |
| Single (%) | 306  (210/96) | 0.19  (0.39) | 0.15  (0.36) | 0.385 |

**Notes:** ‘M’ indicates the mean. ‘SD’ indicates the standard deviation. ^i^ two-tailed p-value from permutation test. p<.05 in bold.
