# Supplementary material for: Can Targeted Intervention Mitigate Early Emotional and Behavioral Problems?: Generating Robust Evidence within Randomized Controlled Trials
Source: PLoS One. 2016 Jun 2;11(6):e0156397. doi: 10.1371/journal.pone.0156397 (PMC4890862; doi:10.1371/journal.pone.0156397)
Supplement: S2 Table — Table A) Impact of PFL on emotional and behavioral functioning—Main and interaction effects controlling for baseline differences. Table B) Impact of PFL on emotional and behavioral functioning—Treatment effects by gender controlling for baseline differences. Table C) Quantile regression results of the distributional impact of PFL on emotional and behavioral functioning controlling for baseline differences. (DOCX) [file pone.0156397.s003.docx]

**S2 Table A**

**Impact of *PFL* on emotional and behavioral functioning – Main and interaction effects controlling for baseline differences**

|  | *UW* | *IPW* |  | *UW* | *IPW* |  | *UW* | *IPW* |
| --- | --- | --- | --- | --- | --- | --- | --- | --- |
| **Panel A: Main Effect Models** | CBCL Internalizing Problems | |  | CBCL Externalizing Problems | |  | CBCL Total Problems | |
| Treatment | 0.30 (1.60) | -0.12 (1.69) |  | 0.58 (1.53) | -0.34 (1.72) |  | -0.24 (1.60) | -1.04 (1.75) |
|  | CBCL Internalizing Problems Cutoff | |  | CBCL Externalizing Problems Cutoff | |  | CBCL Total Problems Cutoff | |
| Treatment | 0.01 (0.04) | 0.01 (0.05) |  | -0.00 (0.04) | -0.01 (0.05) |  | -0.08 (0.04) | -0.10 (0.05)* |
| **Panel B: Interaction Effect Models** | CBCL Internalizing Problems | |  | CBCL Externalizing Problems | |  | CBCL Total Problems | |
| Treatment | 0.75 (2.11) | 0.45 (2.21) |  | 0.93 (2.03) | -0.14 (2.09) |  | 0.63 (2.11) | -0.14 (2.23) |
| Gender (Boys) | 1.22 (2.27) | 2.58 (2.85) |  | 0.59 (2.18) | 1.26 (2.83) |  | 1.47 (2.27) | 2.57 (2.99) |
| Treatment*Gender | -1.23 (3.15) | -1.84 (3.73) |  | -0.85 (3.03) | -0.73 (3.54) |  | -2.12 (3.15) | -2.49 (3.73) |
|  | CBCL Internalizing Problems Cutoff | |  | CBCL Externalizing Problems Cutoff | |  | CBCL Total Problems Cutoff | |
| Treatment | 0.07 (0.06) | 0.09 (0.06) |  | 0.08 (0.05) | 0.07 (0.05) |  | -0.03 (0.06) | -0.06 (0.07) |
| Gender (Boys) | 0.10 (0.06) | 0.13 (0.07) |  | 0.10 (0.05)* | 0.11 (0.06) |  | 0.03 (0.07) | 0.04 (0.10) |
| Treatment*Gender | -0.14 (0.08) | -0.17 (0.09) |  | -0.18 (0.07)* | -0.18 (0.08)* |  | -0.14 (0.10) | -0.14 (0.11) |

Note: n = 163 (intervention 81; control 82). The main effect models reported in Panel A, and the interaction effect models reported in Panel B represent different models. *UW*: Unweighted. *IPW*: Inverse Probability Weighting. Regression coefficients and standard errors (in parentheses) are reported for the continuous scores. Marginal effects and standard errors (in parentheses) are presented for the binary variables. All models control for baseline measures of knowledge of infant development, intentions to use childcare for study child, and use of community services. A logistic model for the Total Problems cut-off score in the interaction analysis could not be estimated as none of the male children in the intervention group reached the cut-off, instead a linear probability model was estimated via OLS. *p<0.05; **p<0.01.

**S2 Table B**

**Impact of *PFL* on emotional and behavioral functioning – Treatment effects by gender controlling for baseline differences**

|  | Boys^a^ | | | | |  | Girls^b^ | | | | |
| --- | --- | --- | --- | --- | --- | --- | --- | --- | --- | --- | --- |
| CBCL Cutoff Scores | *Log.*  *p-value^1^* | *IPW adj. Log.*  *p-*value^2^ | *Perm*  *p-*value^3^ | *IPW adj. perm p-*value^4^ | ES |  | *Log.*  *p-value^1^* | *IPW adj. Log.*  *p-*value^2^ | *Perm.*  *p-*value^3^ | *IPW adj. perm p-*value^4^ | ES |
| Internalizing Problems Cutoff | 0.224 | 0.143 | 0.252 | 0.250 | 0.15 |  | 0.151 | 0.097 | 0.250 | 0.199 | 0.09 |
| Externalizing Problems Cutoff | 0.062 | 0.063 | 0.076 | 0.095 | 0.24 |  | 0.185 | 0.271 | 0.223 | 0.382 | 0.12 |
| Total Problems Cutoff | 0.019* | 0.048* | 0.014* | 0.017* | 0.33 |  | 0.783 | 0.578 | 0.678 | 0.466 | 0.07 |

**Notes:** ^a^ n = 70 (intervention 41; control 29), ^b^ N = 94 (intervention 40; control 54). Perm = permutation test. ‘ES’ = Cramer’s Phi. ^1^ two-tailed p-value from a logistic regression. For boys Total Problems cutoff a LPM model was fitted rather than a logistic regression as treatment status was a perfect predictor of being in the cutoff category. ^2^ two-tailed p-value from a logistic regression (again a LPM model was used for boys Total Problems cutoff) applying inverse probability weights. ^3^ two-tailed p-value from an individual permutation test with 100,000 replications. ^4^ two-tailed p-value from an individual permutation test with 100,000 replications applying inverse probability weights. All models control for baseline measures of knowledge of infant development, intentions to use childcare for study child, and use of community services. * p<.05, ** p<.01 level.

**S2 Table C**

**Quantile regression results of the distributional impact of *PFL* on emotional and behavioral functioning controlling for baseline differences**

| CBCL Quartile | 0.10 | 0.25 | 0.50 | | 0.75 | 0.90 |  | 0.10 | 0.25 | 0.50 | 0.75 | 0.90 |
| --- | --- | --- | --- | --- | --- | --- | --- | --- | --- | --- | --- | --- |
|  |  | **Main Effect Models** | | | | |  |  | **Interaction Effect Models** | | | |
| Internalizing Problems |  |  | |  |  |  |  |  |  |  |  |  |
| Treatment | -0.80  (2.82) | 1.50  (2.41) | 2.00  (1.93) | | -0.06  (2.38) | -2.40  (2.76) |  | -0.80  (3.31) | 1.67  (3.39) | 3.71  (2.73) | -0.50  (3.25) | -1.91  (3.17) |
| Gender (Boys) | ~ | ~ | ~ | | ~ | ~ |  | -1.60  (3.55) | 1.33  (3.64) | 1.71  (2.92) | -0.50  (3.48) | 4.27  (3.40) |
| Treatment*Gender | ~ | ~ | ~ | | ~ | ~ |  | 2.80  (4.93) | -1.00  (5.05) | -2.00  (4.06) | 0.50  (4.84) | -5.82  (4.72) |
| Externalizing Problems |  |  |  | |  |  |  |  |  |  |  |  |
| Treatment | 3.40  (2.35) | 2.00  (2.06) | 1.50  (2.30) | | -4.62  (2.50) | -1.67  (2.78) |  | 2.59  (3.41) | 2.00  (2.69) | 1.47  (2.79) | -5.10  (3.44) | 1.17  (2.73) |
| Gender (Boys) | ~ | ~ | ~ | | ~ | ~ |  | -4.30  (3.66) | -0.18  (2.88) | 0.27  (2.99) | -1.10  (3.69) | 7.67  (2.93) |
| Treatment*Gender | ~ | ~ | ~ | | ~ | ~ |  | 5.22  (5.08) | 0.00  (4.00) | -0.07  (4.15) | 1.55  (5.12) | -8.33*  (4.07) |
| Total Problems |  |  |  | |  |  |  |  |  |  |  |  |
| Treatment | 1.57  (2.02) | 2.57  (1.88) | 1.14  (2.12) | | -1.91  (2.66) | 2.80  (2.52) |  | 2.34  (2.60) | 4.00  (2.45) | 1.78  (2.86) | -1.56  (3.83) | -0.67  (2.42) |
| Gender (Boys) | ~ | ~ | ~ | | ~ | ~ |  | 0.34  (2.79) | 0.33  (2.63) | 0.56  (3.07) | -1.61  (4.12) | 9.00**  (2.60) |
| Treatment*Gender | ~ | ~ | ~ | | ~ | ~ |  | -0.86  (3.87) | -1.33  (3.66) | -1.67  (4.26) | 1.22  (5.70) | -11.00**  (3.61) |

**Notes:** n = 163 (intervention 81; control 82). Coefficients and standard errors (in parenthesis) from quantile regression models. All models control for baseline measures of knowledge of infant development, intentions to use childcare for study child, and use of community services. * p<.05, ** p<.01 level.
